# SILA: a system for scientific image analysis

**DOI:** 10.1038/s41598-022-21535-3

**Published:** 2022-10-31

**Authors:** Daniel Moreira, João Phillipe Cardenuto, Ruiting Shao, Sriram Baireddy, Davide Cozzolino, Diego Gragnaniello, Wael Abd-Almageed, Paolo Bestagini, Stefano Tubaro, Anderson Rocha, Walter Scheirer, Luisa Verdoliva, Edward Delp

**Affiliations:** 1 Department of Computer Science, Loyola University Chicago, Chicago, IL USA; 2 Institute of Computing, University of Campinas, Campinas, SP Brazil; 3 School of Electrical and Computer Engineering, Purdue University, West Lafayette, IN USA; 4 Department of Electrical Engineering and Information Technology, University of Naples Federico II, Naples, Italy; 5 Department of Information Engineering, Electrical Engineering and Applied Mathematics, University of Salerno, Fisciano, Italy; 6 Information Sciences Institute, University of Southern California, Los Angeles, CA USA; 7 Department of Electronics, Information and Bioengineering, Politecnico di Milano, Milan, Italy; 8 Department of Computer Science and Engineering, University of Notre Dame, Notre Dame, IN USA; 9 Department of Industrial Engineering, University of Naples Federico II, Naples, Italy

**Keywords:** Computer science, Information technology, Scientific data, Software

## Abstract

A great deal of the images found in scientific publications are retouched, reused, or composed to enhance the quality of the presentation. In most instances, these edits are benign and help the reader better understand the material in a paper. However, some edits are instances of scientific misconduct and undermine the integrity of the presented research. Determining the legitimacy of edits made to scientific images is an open problem that no current technology can perform satisfactorily in a fully automated fashion. It thus remains up to human experts to inspect images as part of the peer-review process. Nonetheless, image analysis technologies promise to become helpful to experts to perform such an essential yet arduous task. Therefore, we introduce SILA, a system that makes image analysis tools available to reviewers and editors in a principled way. Further, SILA is the first human-in-the-loop end-to-end system that starts by processing article PDF files, performs image manipulation detection on the automatically extracted figures, and ends with image provenance graphs expressing the relationships between the images in question, to explain potential problems. To assess its efficacy, we introduce a dataset of scientific papers from around the globe containing annotated image manipulations and inadvertent reuse, which can serve as a benchmark for the problem at hand. Qualitative and quantitative results of the system are described using this dataset.

## Introduction

Since the early days of photography, images have been used in scientific publications to illustrate the proposed methods, aid in explaining theories, and—most importantly—present the results of experiments. Photography itself became part of experimentation, producing key results such as *Photo 51*, an X-ray diffraction image clearly showing the structure of deoxyribonucleic acid (DNA) for the first time^1^. Later on, with the advent and popularization of digital photography, digital images were added to the scientific repertory, greatly enhancing the speed at which photographic content is produced. In some scientific fields such as biomedicine, images captured by dedicated apparatus are accepted as the results themselves, constituting the elements to be scrutinized while considering a hypothesis^2^.

With the transition from classical photography to digital imaging, editing software entered the scene, allowing researchers to retouch and compose images easily. On the one hand, most of these edits are legitimate, benign, and acceptable. This includes intensity calibrations for visual enhancement or compositions that aim to make the comparison of different outcomes easier. On the other hand, some edits are problematic. Such edits include mistakes (e.g., when the authors inadvertently provide misrepresentations of experimental results) and misconduct (when there is the intent to deceive readers). In this work, regardless of the purpose of a modification under scrutiny, we will call it *image post-processing* since it happens at the end of the digital imaging pipeline, after capture and digitization. Hence, when we uncover image post-processing, we are not claiming it is a mistake or misconduct. We leave such judgments to experts within the scientific fields the images come from.

**Figure 1:**
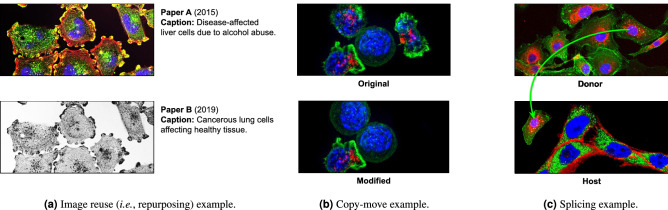
Image post-processing events commonly found in instances of scientific misconduct, which SILA targets. These examples were artificially generated for illustration’s sake. In (**a**), there is a case of image reuse. A culture published in 2015 and claiming liver cells (top) is repurposed in another publication in 2019, claiming lung cells (bottom). In (**b**), there is a case of content copy-move, where the original image on the top is modified to present fewer disease-affected cells (with red spots) at the bottom. Portions of the original content (healthy cell and background pixels) are duplicated to hide and remove undesired cells. In (**c**), there is content splicing. The green arrow points out the spliced cell from the image on the top to the one on the bottom, after rotation and resizing. All original images within this figure are in the public domain^3–6^.

Figure 1 depicts three examples of image post-processing events that might happen in scientific publications, whose detection is tackled in this work. The presence of post-processing events in science is a relevant problem because established cases of mistake and misconduct require that the associated paper be corrected or retracted as soon as possible. Indeed, in addition to the negative impact on a researcher’s reputation, mistakes and misconduct can affect the credibility of scientific research and research outcomes, lead to unfair funding situations, and cause the development of ungrounded techniques if not caught.

In a period of five years (from 2011 to 2015), nearly 78% of the cases of research misconduct identified by the US Department of Health and Human Services, through the Office of Research Integrity (ORI), involved image manipulations^7^. Some cases are reported online^8,9^. More recently, a glut of scientific papers was rushed to publication without proper peer review in an attempt to respond to the outbreak of the 2019 coronavirus disease (COVID-19)^10,11^. Many of these papers gained attention from social media and news outlets, despite containing issues, including inadvertent image post-processing events such as reuse^12^ and duplication^13^. Even with this uptick in cases, the scientific community has been slow to develop solutions to combat this problem systematically.

As a response to the growing problems of mistakes and misconduct related to images in scientific publications, and aware of the importance of performing paper screenings in a faster and more scalable way to deal with large troves of data and reduce the tediousness of the task, we introduce SILA as a system for Scientific Image Analysis. SILA implements a novel human-in-the-loop computational workflow for scientific integrity verification to uncover image post-processing events in scientific publications. It integrates advanced image processing, image forensics, and computer vision solutions to provide meaningful analyses to human experts, helping them to decide if the discovered events are either legitimate or not.

Unlike previous work, the implemented system contains an easy-to-use graphical user interface (GUI) for people outside of computer science and was developed with the aim of easy extension to allow the inclusion of novel image analysis tools quickly. The system requirements were collected with the help of partners from ORI^14^. To test the efficacy of the proposed workflow and the implemented system, we conduct experiments and report the performance of each component of the solution over a carefully collected and annotated dataset containing real cases of retracted papers with questioned images.

In summary, the contributions of this article are:

- A unique and principled combination of advanced image processing, image forensics, and computer vision tools, such as copy-move detection and provenance analysis, custom-tailored to the problem of uncovering image post-processing events in scientific publications. To the best of our knowledge, this is the first time image provenance analysis is applied to the problem at hand. In a nutshell, provenance analysis aims at examining multiple images with the intent to ascertain their shared history of edits, expressing through a graph how one image might have given rise to the other, even if they come from different publications.
- A system (namely SILA) that implements an end-to-end workflow—from Portable Document Format (PDF) files to image provenance graphs—to help experts in (i) spotting image post-processing events and (ii) deciding how legitimate they are. While most tasks are automated (such as image and caption extraction from the binary stream of PDF documents), the ultimate decision-making is left to the experts, who have the final word based on the provided evidence.
- A new dataset containing both retracted (due to the documented presence of image post-processing events) and so far unchallenged scientific papers, which we are making available to the community, in the hope that it becomes a standard benchmark for algorithm and system development. This dataset has 988 publications from different countries. While it is smaller than others in the literature^15–17^, it provides a more complete set of annotations and task-specific metrics that will help future work that uses it as a benchmark.

## Results

Outcomes of this work are herein organized in terms of (i) the resulting implemented system, (ii) the dataset of papers used in the experiments and qualified for becoming a scientific integrity benchmark, (iii) qualitative results demonstrating some of the system’s capabilities over this dataset, and (iv) quantitative results of the system’s capabilities over this dataset.

### Implemented system

**Figure 2:**
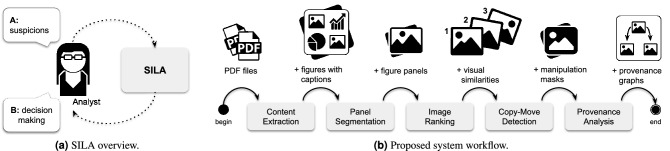
The SILA system. In (**a**), the devised operation of SILA, highlighting its human-in-the-loop aspect. An analyst who has suspicions about a set of scientific publications feeds them to the system as PDF files. The system then performs a series of content analyses, which provide evidence to allow the analyst to make a decision about the publications’ integrity. In (**b**), the details of the proposed series of analyses (system workflow), composed of five tasks (depicted as rounded rectangles) with their respective outcomes (preceded by a “+” sign, to indicate their addition to the system final output).

Figure 2a provides a high-level overview of the devised operation for SILA. It highlights the human-in-the-loop nature of the system, which supports an analyst who has suspicions about a set of scientific publications. The system feedback is provided under the analyst’s demand, who can gradually gather evidence on the integrity of the publications’ images before deciding whether they represent misconduct.

Figure 2b details the sequence of the five tasks that an analyst can perform over SILA, illustrating the proposed system workflow. As one might observe, each task generates a particular type of information (e.g., extracted figures with their respective captions, masks highlighting the manipulated image regions), which is added to the system’s final output repertoire. The five implemented tasks are:

- *Content extraction* This is the task responsible for automatically extracting figures and their respective captions from the given suspect PDF files. Images are decoded from the PDF binary stream of objects, and captions are retrieved from the PDF text before being associated with their respective images. Captions play an important role in the understanding of the context of published figures.
- *Panel segmentation* Many figures from scientific papers consist of multiple panels^18^. This task allows the analyst to select multi-panel figures (for instance, figures composed of one or two outputs of microscopy, graphs, and bar charts collated together) and automatically segment them into smaller parts, one for each constituent panel. By doing this, the system helps to eliminate the irrelevant background and prioritize analyses on each panel individually.
- *Image ranking* Once images extracted from the PDF files are made available (including both the original entire figures and their respective panels after the proper segmentation), analysts are allowed to select, as many times as they want, an image of interest and retrieve similar content existing across the various PDF files, such as exact copies, near-duplicates, and semantically similar elements, in a similar fashion to Google reverse image search^19^. Image ranking helps to reduce the clutter and allows the analyst to focus on a particular set of potentially related figures.
- *Copy-move detection* This task allows the analyst to perform a single-image analysis over a selected figure (or even a selected panel, after the proper segmentation). The aim is to inspect the image for cloned regions (i.e., copy-move detection^20^), which might be a strong indication of either the fabrication or the hiding of unwanted results.
- *Provenance analysis* In addition to inspecting single images, provenance analysis is made available to the analyst as a novel tool to detect both content splicing and reuse across different sections of one or more publications. Briefly, provenance analysis^21^ provides explanations for how sets of figures and figure panels may be sharing visual content, depending upon the selection of the analyst.

**Figure 3:**
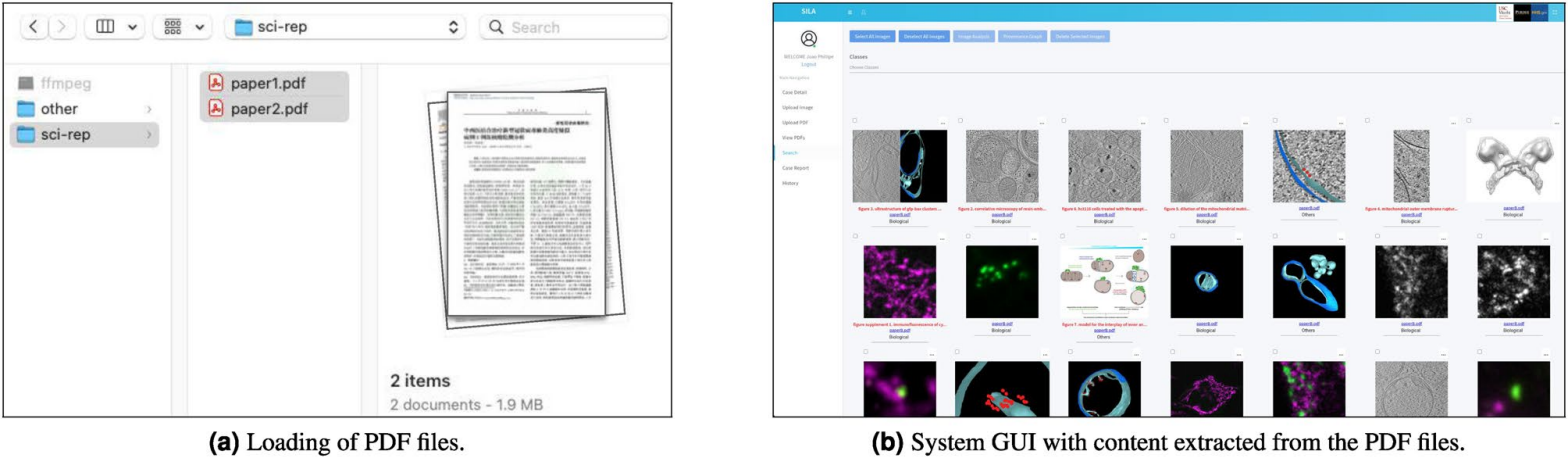
Examples of the implemented system GUI components. In (**a**), the operating-system agnostic interface allows the analyst to upload multiple scientific papers of interest as PDF files. In (**b**), the web browser-based system GUI is already populated with content automatically extracted by the system from the provided PDF files. The interface follows the standard of web pages, letting the analyst perform each one of the five tasks represented in Fig. 2b. In this example, for illustration’s sake, the depicted content was extracted with SILA from the publication available through the DOI 10.7554/eLife.40712, under the Creative Commons public domain license.

Figure 3 shows an example of the system GUI, whose aim is to provide an intuitive interface that most people are familiar with. Hence, we have implemented a web-based GUI containing rich input and output graphical components (such as web pages, weblinks, click buttons, selection boxes, image panels, etc.). These components can be quickly rendered on the client-side by any modern web browser, with no need to install specialized software by the analyst. To use the system, all they need to do is access the web address of the application.

Given the sensitive application domain and the capabilities of SILA, we have decided not to make it unrestrictedly publicly available online. The main reason for this is to avoid the unruly usage of the system to indiscriminately accuse professionals of practicing scientific misconduct without the proper human expert supervision and the assurance of the right of defense. Consequently, we will make a fully-coupled version of SILA only available to properly identified parties upon formal request and the sign of a responsibility agreement. Regardless of that, we are setting the decoupled modules of the system up on GitHub (see https://git.io/JwWVe) as our best effort to keep this work scientifically reproducible by the community.

### Scientific papers dataset

The Scientific Papers (SP) dataset is a set of scientific publications containing samples collected from all over the world with the documented presence of image post-processing events. To gather these samples, we first started by selecting 298 retracted publications from hundreds of journals, whose retractions occurred due to problems with images such as duplications, fabrications, manipulations, and reuse. We then focused on each paper’s corresponding author to collect more of their co-authored publications, including any other retracted, corrected, or regularly published papers. By doing so, we gathered an additional 358 papers. In anticipation of the need for assessing false-alarm rates (when targeted conditions are detected even though there are no documented problems), we also collected a set of 332 biomedical papers from PubMed Central^22^ without known issues. Each paper contained at least one published image whose findings have not been publicly implicated in any integrity investigation. All the selected publications were identified using only publicly available information. Inclusion in the dataset does not necessarily signify that an article is the subject of any research misconduct allegation or proceeding.

We are making this dataset available to the community (see https://git.io/JcZsX), along with some task-specific ground-truth annotations (e.g., cloned regions within the manipulated images, provenance graphs detailing reused images across papers). Each paper is referred to by its Digital Object Identifier (DOI) and, depending on the availability in the publisher’s website, we provide links to information such as publication webpage, PDF file, figures, figure captions, all encoded as publication-wise metadata in JavaScript Object Notation (JSON)^23^.

**Figure 4:**
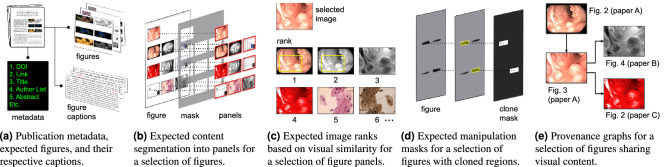
Provided annotations within the SP dataset. In (**a**), the additional data and annotations related to the task of content extraction. Metadata about the publications and links to the original paper figures supplied by the publishers’ websites are provided, along with their respective captions. In (**b**), the annotations related to the task of panel segmentation. For a set of selected paper figures, manually annotated content masks are provided, defining multiple bounding boxes that individually encompass panels of interest for further analyses. In (**c**), the annotations related to the task of image ranking. A set of selected figure panels is provided, with each panel containing its list of visually similar images (i.e., image rank), sorted from the most to the least similar element. In (**d**), annotations related to the task of copy-move detection. For a set of selected paper figures, manually annotated masks are provided, highlighting the cloned regions within each image. In (**e**), annotations related to the task of provenance analysis. Provenance graphs are provided in JSON format, linking a selection of figures that share visual content and are reused across a subset of the dataset papers. Original images within this figure are in the public domain^3–6,24–26^.

Figure 4 summarizes the annotations supplied for each one of the tasks depicted in Fig. 2b. For the task of content extraction, we provide links to 1876 original figures in high resolution, which were made available by the publishers’ websites for a subset of 285 articles of the dataset. These figures are accompanied by the text of their captions within their respective manuscripts. This content aims at being a ground truth for solutions designed to extract images and captions from PDF files.

For the task of panel segmentation, we recruited two annotators to manually segment the same 303 figures, all provided by the publishers’ websites of 48 articles, which were randomly selected from the dataset. To check the agreement level of the individual panel annotations, we used the Intersection over Union (*IoU*) score, which is calculated as the respective pixel-wise intersecting panel areas divided by their union. As a consequence of this definition, *IoU* values fall within the [0..1] interval, where strong agreements lie closer to one. For the case of the two annotators, the image-wise average *IoU* score was 0.88, indicating a significant amount of agreement between them. With redundant annotations, we decided to focus on the annotators’ agreement to constitute the final ground truth for the 303 figures. We thus used the overlapping areas between both annotations to generate the offered set of expected panel segmentation masks.

For the task of image ranking, we extracted and selected 2843 figure panels from the PDF files of 48 articles of the dataset, and asked two annotators to establish ranks for each one of these images individually. Each panel-wise rank was thus built from the remaining 2842 available panels and contained, according to the opinion of each person, the up-to-ten most similar images in terms of type (e.g., microscopy, western blots, graphs), texture, and color. As a result, the two independent annotators produced ranks that presented an average difference of 4.3 panels (the largest possible difference was 20 panels, when the annotators completely disagreed with each other), with a standard deviation of 3.6 when compared pairwise, considering all of the 2843 cases. To generate the final ground truth for each image, we took the union of the respective annotators’ ranks.

For the task of copy-move detection, we manually selected 180 figures from 125 papers of the dataset, which are flagged, according to their respective retraction notices, as depicting cloned regions. We then recruited six volunteers to go through the retraction notices and manually annotate the described cloned regions. We asked three folks to annotate each figure and adopted the intersection of the provided annotations as the final manipulation mask. As a result, we obtained a set of 180 pixel-wise copy-move manipulated region masks, which we are making available to the community (see https://git.io/JKltM).

Finally, after a throughout inspection of the retraction notices available on the dataset, we selected 85 significant cases of figure reuse across different publications, which are helpful to the task of provenance analysis. For these cases, we analyzed the retraction documents and manually tracked down the figures that share visual content. As a result, we established 70 unique provenance graphs, whose nodes individually represent a scientific figure with its publication source, and whose edges explain how one publication has possibly reused the content of another, connecting from the older to the newer article. We are making these graphs available to the community (see https://git.io/JKidk) in the JSON format proposed by Yates et al.^27^

Here, it is essential to clarify that it is not our aim with the SP dataset to ultimately classify the constituent papers as “pristine” versus “problematic”. The statuses of the papers in this regard are very dynamic, depending on long and meticulous investigations that might either happen or be contested in the future. Such a sensitive task is out of the scope of this work.

### Qualitative results

Here we present qualitative results that illustrate how the proposed system behaves in the face of (i) cloned content within single images (through copy-move detection) and (ii) image reuse across multiple papers (through provenance analysis).

#### Copy-move detection

To analyze images individually and look for post-processing events, we propose to rely on copy-move detection. Copy-move operations comprise post-processing events in which a region of an image is cloned somewhere else within itself^20^, usually with the intent to cover an undesired feature, by replicating either an existing object (duplication) or background pixels (removal).

**Figure 5:**
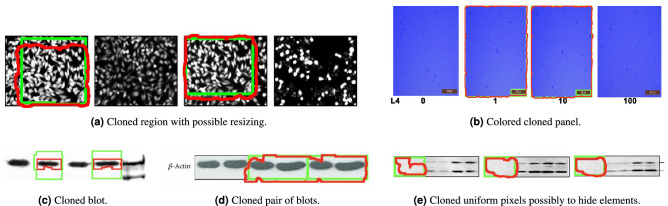
Results of copy-move detection that agree with existing retraction notices. In green boxes, the manual annotations provided as ground truth within the herein introduced SP dataset. In red, the cloned regions identified by SILA. The system correctly identifies cloned image regions in different scenarios. In (**a**), microscopy images of incubated cells. In (**b**), cell cultures that were captured by an inverted microscope. In (**c**–**e**), western blots. When SILA points out a cloned region, it does not necessarily mean a mistake or misconduct. All original images within this figure belong to papers published as Creative Commons content. The respective sources are available through DOI 10.1074/jbc.M109.090209, 10.1371/journal.pone.0085808, 10.1186/1471-2180-6-26, 10.1155/2014/987017, and 10.1074/jbc.M302674200. Here, they were cropped, enlarged, and annotated over, in either green or red, to convey the findings of SILA.

**Figure 6:**
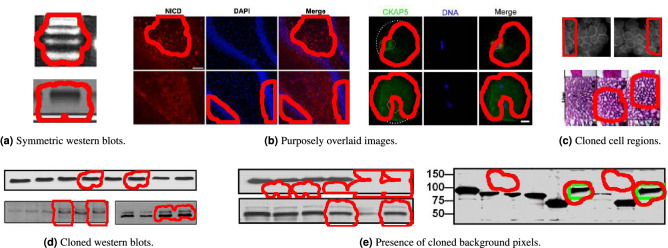
Results of copy-move detection that are not mentioned in existing retraction notices. Regions with red borders depict the cloned content found by SILA. Many of these regions do not present a respective ground-truth green box because they have never been acknowledged in a retraction notice. In (**a**), cloned regions detected due to the perfectly symmetric nature of some western blots. In (**b**), cloned regions detected due to panel overlays. In (**c**), cloned regions over microscopy outputs that might have been difficult for a human reviewer to spot in the past. In (**d**), more of these hard-to-spot regions, this time on western blots. In (**e**), cloned background pixels, among others. When SILA finds a cloned region, it does not mean there was necessarily a mistake or misconduct. Examples in (**a**) and (**b**) are probably legitimate and highlight the importance of human intervention when deciding if cloned content is acceptable or not. Analysts have probably overlooked the issues depicted in examples (**c**–**e**). All original panels within this figure belong to papers published as Creative Commons content, under DOI (**a**, top) 10.1074/jbc.M806041200, (**a**, bottom; **d**, bottom) 10.1074/jbc.M111.255042, (**b**, left; **e**) 10.1074/jbc.M808084200, (**b**, right) 10.18632/oncotarget.15097, (**c** top; **d** top) 10.1074/jbc.M111.274613, and (**c** bottom) 10.3389/fphar.2016.00226. Here, they were cropped, enlarged, and annotated over, in either green or red, to convey the findings of SILA.

In Fig. 5, we show some of SILA’s results that are aligned with the human annotations provided within the SP dataset. Green boxes highlight the ground truth provided by annotators, while the red regions were automatically obtained through the system. As one might observe, SILA can detect copies coming from different panels of the same figure. It can deal with both gray-level and colored images (see Fig. 5a,b), and even with scientific imagery such as western blots (see Fig. 5c–e). In the latter case, SILA was able to detect the replication of small areas, including cloning possibly related to content removal (see Fig. 5e). Of course, these analyses do not necessarily point out malicious manipulations. Instead, they aim at helping analysts focus on suspiciously similar content and rule the outcome by themselves.

To gain insights into the complexity of the task at hand and the need for human intervention, we show some interesting SILA results in Fig. 6. In Fig. 6a, for instance, there are bilaterally symmetric western blots that may cause false alarms to our detector. SILA recognizes two symmetric regions in the image, but this does not mean manipulation was carried out since the algorithm cannot distinguish between fabricated mirrored areas and authentic symmetric patterns. Another common situation is presented within Fig. 6b. It often happens that, in a timed experiment, the same substrate is imaged several times and under different conditions, to ultimately be either compared side-by-side or overlaid to generate a richer representation. This is the case of the two rightmost “Merge” panel columns depicted within Fig. 6b, which are indeed a combination of their respective previous panels (therefore sharing similar regions). SILA can detect these replications, but their existence is legitimate and would not be considered evidence of manipulation by a human analyst.

Lastly, Fig. 6c–e depict examples of suspect cases that were probably overlooked by human analysts. Many of these images were not previously acknowledged as containing duplicated content. Nonetheless, SILA can spot cloned content within them (see Fig. 6c,d), and even probable content removal (see Fig. 6e, where uniform background pixels might have been used to make blot lanes clear). It is worth mentioning that all of these figures came from already retracted papers, and only one of them was previously identified by a person as containing a duplication (see the pair of green boxes within the rightmost panel of Fig. 6e). All the remaining issues passed unnoticed to the human annotators but not to SILA.

#### Provenance analysis

**Figure 7:**
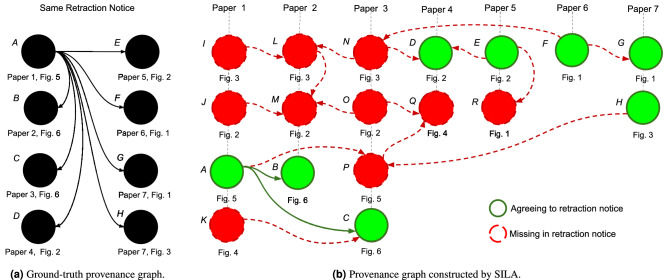
Result example of provenance analysis applied over a particular case of inadvertent image reuse across seven different papers, which are part of the SP dataset. In (**a**), the provided annotation (ground-truth provenance graph), obtained through a manual curation of the content of the retraction notice available under DOI 10.1371/journal.pone.0190562. According to this document, Fig. 5 from Paper 1 (node *A*) shares elements with other seven figures coming from six distinct papers (herein represented by nodes *B*-*H*). In (**b**), the resulting provenance graph computed by SILA. Besides finding the expected images of nodes *A*-*H* (highlighted in green, to show agreement with the retraction notice), SILA also encountered other ten figures presenting evidence of content sharing (represented by nodes *I*-*R* and herein highlighted in dashed red, to indicate absence in the retraction notice).

**Figure 8:**
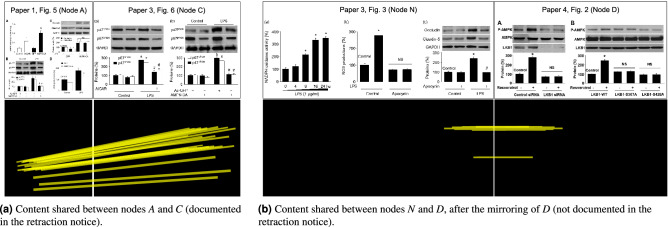
Details of two edges computed by SILA within the provenance graph depicted in Fig. 7b. In (**a**), the depiction of the shared content between nodes *A* and *C*, whose relation is documented in the respective retraction notice. In (**b**), the depiction of the shared content between nodes *N* and *D*, after a mirroring operation over *D*. Image *N* is not identified as sharing content within the retraction notice, suggesting the relation *N*-*D* was not found by a human analyst before. The four top panels herein presented belong to the following papers: (i) “Paper 1, Fig.  5”, reprinted from Experimental and Molecular Pathology, 97, Zhao et al., “Activation of AMPK attenuates lipopolysaccharide-impaired integrity and function of blood-brain barrier in human brain microvascular endothelial cells”, 386-392, 2014, with permission from Elsevier; (ii) “Paper 3, Fig. 6” and “Paper 3, Fig. 3”, “Activation of AMPK improves lipopolysaccharide-induced dysfunction of the blood-brain barrier in mice”, Yu et al., Brain Injury, March 3, 2015, reprinted by permission of the publisher, Taylor & Francis Ltd; and (iii) “Paper 4, Fig. 2”, “Resveratrol via activation of LKB1-AMPK signaling suppresses oxidative stress to prevent endothelial dysfunction in diabetic mice”, Hu et al., Clinical and Experimental Hypertension, May 5, 2016, reprinted by permission of the publisher, Taylor & Francis Ltd. The bottom row panels represent the masks of the respective top images, with the respective positions of duplicated content found by SILA linked through yellow lines. By indicating here the duplicated content, we are not claiming this is the outcome of a mistake or misconduct.

In addition to inspecting single images with copy-move detection, SILA allows the usage of provenance analysis to investigate how sets of images may be sharing visual content. This approach serves to detect both content splicing and reuse across different sections of one or more publications. As explained by Moreira et al.^21^, provenance analysis aims at generating, for a given set of images, the directed acyclic graph (DAG, namely provenance graph) whose nodes individually represent each image of the set and whose edges express the most probable edit and content-donation history between pairs of images. The edits can include cloning, cropping, blurring, and splicing, linking seminal elements to derived elements.

Figure 7a depicts a provenance graph representing a confirmed case of image reuse across different scientific papers, which was manually constructed based on the content of one of the retraction notices belonging to the SP dataset. As we have mentioned before, we are making 70 of these ground-truth graphs available as special annotations of the dataset. Figure 7b depicts the result of provenance analysis performed by SILA over all the 45 figures belonging to the seven papers involved in the reuse case described in Fig. 7a. Coincident nodes and edges between the ground-truth graph and the provenance graph computed by SILA are highlighted through green lines. In dashed red lines, the remaining content represents material detected by SILA that was not reported in the retraction notice (hence comprising undocumented and potentially unknown issues). As one might observe, SILA correctly identified all the documented reused figures (represented by nodes *A*-*H*). Moreover, the other ten figures (*I*-*R*), out of 45, were also identified as relevant for further human examination.

Figure 8a details the shared visual content identified by SILA between nodes *A* and *C* (already described in the retraction notice). Figure 8b, in turn, details the shared content between node *N* (unknown to the retraction notice and the SP dataset manual annotations) and node *D*. SILA was able to spot their similarity, despite one content being a mirrored version of the other. This indicates that the extra nodes found by SILA are not false alarms but undocumented cases of reused content. We reiterate that by identifying duplicated contents in these experiments, we are not claiming they are the effect of mistake or misconduct. Authors might have reasonable explanations for such occurrences.

### Quantitative results

We now report results of SILA over the SP dataset for each one of the tasks implemented in the system and annotated in the dataset. Each task has its own suggested metric (or set of metrics), whose SILA performance values we are providing to serve as a baseline for future research on scientific image integrity analysis.

Table 1 summarizes the results of SILA. The first two columns are related to the task of content extraction. To evaluate the quality of image extraction, we used SILA to extract figures from a subset of 285 PDF files of the SP dataset, which contained the original images provided by their respective publishers’ websites. The idea was to report the fraction of the curated figures successfully obtained by SILA, namely the system image recall (*IR*). As one might observe, SILA *IR* was equal to 0.71. Considering that respective original and extracted figures often present differences in resolution and translation (since the original figures are usually provided in the publisher’s website at a higher resolution, with differences in the outside borders and cropping place), SILA was able to successfully and automatically extract 71% of these images. To mitigate the cases where SILA fails to extract a target figure but the analyst is still able to get the source image directly with the authors or the publisher of the paper, we added to the system an “upload figure” function, where users can also ingest image files to the workflow.

Besides figure extraction, SILA also obtains figure captions from the PDF files, which are automatically linked to their respective extracted images. To assess the quality of caption extraction, we measured the integrity of the output captions by computing two metrics. Namely, (i) normalized Levenshtein distance (*LD*)^28^, which aims at verifying if the extracted captions match the ground truth at the character level, and (ii) BERTScore (*BS*)^29^, which aims at assessing the semantic similarity between captions by using contextual word embeddings. Both metrics lie in the [0..1] real interval, but contrary to *BS* (a similarity measure), we want *LD* as small as possible since it is a distance. SILA obtained an average of $LD=0.10$ (close to zero, as expected) and an average of $BS=0.88$ (close to one) over the 1874 annotated captions of the SP dataset.

The third column of Table 1 is related to panel segmentation. To measure the quality of the segmentation of compound figures, we used the intersection over union score (*IoU*), which is computed by comparing the ground-truth segmentation masks and the respective masks generated by SILA, at the pixel level (see Fig. 4b for a mask example). *IoU* also lies in the [0..1] interval and we want it as large as possible. On average, SILA agreed on 48% of the mask pixels of the SP dataset, considering 303 figures. This is certainly one of the system capabilities that deserve attention in the future, but the current performance is not disabling, since SILA can execute the other tasks on either the sub-panels, if available, or the entire figures.

The fourth column is related to the task of image ranking. For this one, we suggested using the precision at the top-*N* retrieved images (*P*@*N*, with $N \in \{1, 5, 10\}$), averaged over the 2843 figure panels with curated ranks of the SP dataset. In a nutshell, *P*@*N* is the fraction of the top-*N* retrieved images (for a given image of interest) that are relevant (i.e., they belong to the ground-truth image rank). *P*@*N* lies in the [0..1] real interval, and we want it as large as possible. In the case of SILA, on average, 59% of the top-one retrieved figure panels by the system were relevant, indicating room for further improvements but already useful to provide clues of content reuse to be followed by the analysts.

**Table 1:** Quantitative results of the tasks performed by SILA.

Image extraction		Caption extraction		Panel segmentation		Image ranking		Copy-move detection		Provenance analysis	
*IR*	0.71	*LD*	$0.10 \pm 0.22$	*IoU*	$0.48 \pm 0.19$	*P*@1	$0.59 \pm 0.49$	$F_1$	$0.35 \pm 0.30$	*VO*	$0.64 \pm 0.15$
		*BS*	$0.88 \pm 0.24$			*P*@5	$0.41 \pm 0.32$			*EO*	$0.16 \pm 0.32$
						*P*@10	$0.32 \pm 0.24$			*VEO*	$0.50 \pm 0.19$
1876 figures		1874 captions		303 figures		2843 panels		180 figures, 180 masks		591 figures, 70 graphs	
285 papers		285 papers		48 papers		48 papers		125 papers		85 papers	

All values belong to the real interval [0..1]. For all metrics but *LD*, the larger the value, the better the solution. All values are averaged over the sets of elements in the bottom row (± deviations), except for *IR*.

Metrics: *IR*—image recall, *LD*—Levenshtein distance, *BS*—BertScore, *IoU*—intersection over union, *P*@*N*—precision at top-*N* images, $F_1$— $F_1$-score, *VO*—vertex overlap, *EO*—edge overlap, *VEO*—vertex and edge overlap.

The fifth column of Table 1, in turn, is related to copy-move detection. We developed a new copy-move detector to add to SILA, which is specialized in scientific imagery. To evaluate this new detector, we averaged the pixel-wise $F_1$-scores ($F_1$) between the 180 ground-truth cloned-region masks manually annotated in the SP dataset and their respective clone masks produced by SILA (see Fig. 4d for a mask example). $F_1 \in [0..1]$ is a metric that expresses the harmonic mean between the recall and the precision of in-agreement pixels between ground-truth and system-computed clone masks, with ideal masks presenting $F_1$ close to one. SILA’s performance was $F_1=0.35$ on average, which is the state-of-the-art result for scientific images, as we detail in the supplementary material S1 that accompanies this manuscript. As we have shown in the previous section through qualitative examples, the ground truth provided by our team often comprised laxly annotated boxes, which included pristine background pixels (see green boxes in Fig. 5c, for instance). In these cases, SILA was more precise than the ground-truth annotations, hence being penalized for deviating from them. Indeed, the $F_1$-score of SILA’s mask over Fig. 5c was only 0.48, despite correctly finding all the cloned blots. For the undocumented cases of Fig. 6c,d, the $F_1$-scores were zero, regardless of their relevance for further human analysis. This indicates that the annotations provided within the SP dataset can still be enhanced in the future, hopefully with the help of an engaged scientific community in the herein introduced benchmark.

Lastly, the sixth column is related to provenance analysis. To assess the quality of the provenance graphs computed by SILA, we followed the experimental setup proposed by Yates et al.^27^. We thus report the average vertex overlap (*VO*), edge overlap (*EO*), and vertex and edge overlap (*VEO*) of the 70 computed graphs when compared to the ground truth introduced in the SP dataset, ignoring the direction of the edges (undirected *EO* and *VEO*^21^). As discussed by Papadimitriou et al.^30^, these metrics quantify the overlap between the ground-truth and the respective solution-provided graphs through $F_1$-scores of the retrieved elements. As a result, they all lie in the [0..1] interval and should be as close to one as possible. Due to the more challenging nature of the SP dataset (which contains mostly scientific images, as opposed to typical natural scenes), the SILA provenance results are not as good as other results from the literature^21,31,32^, which used natural images. Anyway, the graphs generated by SILA are already useful to put image reuse in evidence, as one might observe through Fig. 7b. In such case, the provenance metric values were $VO=0.64$, $EO=0.16$, and $VEO=0.50$, mainly harmed due to the presence of undocumented figure reuse, such as the one represented by node *N* (see Fig. 8b). Similar to the case of copy-move detection, annotated provenance graphs can be improved in the future by benefiting from the community’s engagement with the benchmark.

## Discussion

The qualitative results demonstrate that SILA has surpassed the scientific peer reviewers’ capability of spotting image post-processing events, in the specific cases of the herein mentioned publications. They also indicate that the system can perform the automatic finding and principled documentation, through cloned-region maps and provenance graphs, of enough evidence to support an analyst’s determination on whether or not image post-processing events are legitimate. The quantitative results, in turn, show that there is indeed still room for future improvements and more examination. Nevertheless, the reported numbers are in accordance with establishing the very first baselines for a number of tasks, which in the future might receive attention from the scientific community, towards the development of new methods and the improvement of the annotations of the SP dataset, as its content is more and more scrutinized. We also believe SILA is the first step to make analysts start moving from simply finding out *enough evidence* for retraction towards an *exhaustive search* for existing problems. We sincerely hope other researchers will join us in this endeavor.

Previous work has studied the topic of scientific image post-processing. Rossner and Yamada^33^ early described the temptation of manipulating images for scientific publication, due in part to the availability of editing tools such as Adobe Photoshop. They outlined hypothetical image manipulations problematic from a biological research standpoint, whose detection is worth pursuing when developing tools to verify integrity. Also aware of the existence of such manipulations, Cromey^34^ proposed 12 guidelines to guide authors and publishers to either accept or question image post-processing practices.

Aiming at getting a sense of the frequency of the problem, some works collected and manually analyzed scientific papers, looking specifically for image reuse. Oksvold^35^, for example, collected a total of 120 papers from three journals related to cancer research, to manually analyze their images with the help of Adobe Photoshop. By zooming in on high-quality images obtained from the papers and comparing them side-by-side, Oksvold concluded that nearly 24% of the articles contained images used to represent the outcome of two or more different experiments. Similarly, Bik et al.^15^ manually screened 20,621 papers in 40 scientific journals, using color adjustments and visual comparison in Apple Preview. With this approach, they claimed the identification of 782 papers (3.8%) containing duplicated images.

Aware of the importance of performing screenings faster and more scalable, some works in the literature aimed at the automated analysis of scientific papers. Bucci^16^, for instance, proposed a method to automatically extract images from PDF files by converting each PDF page to a single digital image. Over these page images, he proposed the application of image processing morphological operations to segment the visual content into meaningful graphical elements (e.g., figure panels, such as charts, western blot bands, etc.) for further analysis. Panels were then automatically inspected for gel-electrophoresis fabrication (through the measurement of suspicious background pixel intensity discontinuities) and content duplications (through a third-party software solution). With this approach, Bucci analyzed 4778 panels extracted from 1364 papers published in 451 biomedical journals, finding out that 5.7% of the articles presented manipulations.

Similarly, Acuna, Brookes, and Kording^17^ investigated the application of techniques from computer vision and machine learning to select suspect images from a collection of source figures published along with a set of questioned papers. From computer vision, they used interest-point matching and clustering to spot cloned content between figures. From machine learning, they used gradient boosting to classify images as being either biomedical (therefore of interest) or non-biomedical (such as chart labels or indicative arrows, hence not of interest). Selected images were then presented to human experts for a final decision. The authors evaluated nearly 2.6 million images belonging to 760,036 articles obtained from 4324 biomedical journals with such an approach. Three analysts found out that 1.47% of these articles contained duplicated images. The list of analyzed articles is sensitive and not freely available to the public due to legal aspects.

Some works relied on techniques from image forensics^36^ to perform integrity verification. Farid^37^, for example, presented early digital integrity verification solutions to localize content removal and duplication within single scientific images. Toy examples were used to show the efficacy of the algorithms. More recently, Xiang and Acuna^38^ introduced methods to suppress scene content and extract residual features over single scientific images, emphasizing the noise patterns that occur due to manipulation artifacts. The researchers downloaded and purposely doctored scientific images from the web to train and test their data-driven approach, such as microscopy and western blots. As a result, they obtained a dataset containing nearly 750 curated images. Finally, Mazaheri et al.^39^ designed a deep learning approach to detect image duplications. Similar to SILA, they also proposed a workflow that starts with the paper PDF files, but their chosen focus was on classifying manuscripts as either containing duplications or not, rather than providing different types of evidence.

Last but not least, some researchers have also tried to create helpful image datasets for scientific integrity verification. More notably, Koker et al.^40^ recently introduced a set of near-duplicate scientific images, purposely manipulated to try to reproduce real cases of inadvertent image reuse on publications. In opposition to producing a synthetic dataset, the HEADT Centre^41^ has just started to collect and structure a dataset containing real-world images from retracted scientific publications. At the current stage, though, this dataset still lacks reviewed and rich annotations, such as clone maps and provenance graphs.

Despite the invaluable previous efforts of the scientific community, the problem of scientific image integrity verification is still open, growing in worldwide occurrence and relevance, and still needing attention. In particular, the lack of a unified benchmark that allows the principled evaluation of different techniques hinders the advance of the state of the art. Currently, it is not easy for researchers to either compare or reproduce each others’ results. To the best of our knowledge, there is also no large dataset freely available out there containing diverse, well-documented, and confirmed cases of image manipulations, with rich annotations that are precise enough to allow the calculation of objective metrics. We believe this publication takes a fundamental first step in such direction by addressing these aspects.

Moreover, SILA implements the first human-in-the-loop end-to-end workflow that starts with PDF files and gradually adds new evidence to the system output, to support the analyst’s final decision. It does not aim at replacing humans but at saving their time by making tedious tasks more precise and automatic. It counts on a novel copy-move detector, which was fine-tuned to the case of scientific images, and on provenance analysis, which was used for the first time to process scientific images. We believe cloned-region maps and provenance graphs, respectively the outputs of copy-move detection and provenance analysis, would bring a huge benefit if added to retraction notices, by making them much more understandable.

### Limitations and future work

The herein addressed issues with scientific images do not comprise an exhaustive list of problems. The proposed and discussed capabilities of SILA are focused on the five tasks depicted in Fig. 2b. The system and the introduced annotated dataset lack representation and mitigation of other problems, which we expect to address collaboratively with an engaged community in the future. For instance, SILA currently misses the detection of (i) content removal that does not involve cloning background pixels to hide foreground, and (ii) content splicing whose donor images (see Fig. 1c) are not available. In these situations, neither copy-move detection nor provenance analysis can provide evidence. It thus remains to further SILA extensions the addition of single-image inspection solutions supported by noise analysis^42^. Another recent technology from our repertoire yet to be added is the detection of synthetically generated images, such as synthetic western blots^43^, which might be used to forge the outcome of ungrounded experiments. Lastly, SILA does not directly read yet images stored in Tag Image File Format (TIFF) containers, a feature presently under development.

## Methods

To implement the SILA workflow, we developed a client-server software architecture that aimed to give the analysts the freedom to concurrently execute their duties on the system, with no interference of one’s analyses into the others’. To make the system extensible, we implemented each task from the proposed workflow as an independent *forensic container*, a standalone executable piece of software that contains everything needed to run it, including libraries, configurations, and even specific operating-system environments. By using tools such as Docker^44^, novel and better forensic tools can be easily added to the system, as long as they are bundled as a container.

Concerning the task of content extraction, we used MuPDF^45^—an open-source software library developed to parse PDF streams—to read the cross-reference (*a.k.a.* Xref) tables of a given PDF document, and extract the indexed and stream-embedded images. To gather text, we relied on PDFMiner^46^, another open-source Python PDF parser, which is better suited to extract figure captions. To facilitate reproducibility, we are making this implementation and image-extraction experimental setup available to the community at https://git.io/JcZGM.

For the task of panel segmentation, we chose the data-driven solution introduced by Tsutsui and Crandall^47^. As explained by the authors, a data-driven approach for multi-panel image segmentation has the potential to be more tolerant to the diversity of image composition layouts one may find in scientific papers if properly trained with enough examples. They defined the segmentation problem as an object detection variety whose purpose is to predict the bounding boxes of the individual panels. The prediction process was learned with a convolutional neural network, namely the YOLOv2 system^48^. The herein used Tsutsui’s implementation and trained models are available online^49^. For reproducibility sake, we are making this experimental setup available at https://git.io/JcZG2.

For image ranking, we relied on one of the fastest methods to detect interest points, namely SURF^50^. Therefore, we detect the 500 most important interest points within a given image, according to the corner-relevance Hessian value inherent to SURF. To describe the interest points, we adopted the RootSIFT^51^ descriptor, a variation of SIFT^52^ that is reportedly better suited to image retrieval. Although this approach is invariant to rotation and scale image transformations, it does not cope with mirroring. Hence, we additionally detect and describe 500 interest points over a horizontally mirrored version of the given image. Consequently, each image of interest is mapped to a set of 1000 128-dimensional RootSIFT feature vectors, which are then used to build the image index. Consider analysts work on a one-case-at-a-time basis, where dozens of PDF documents belonging to a set of collaborating authors are put together to constitute a case. Dozens of PDF documents will commonly lead to hundreds of image panels, which will thus lead to less than one million RootSIFT feature vectors per case. This quantity poses no challenge to the state-of-the-art feature-indexing technology and implementation^53,54^. Hence, we build a flat *l*2-distance-based inverted-file index that contains all the case images for each case. As a result, whenever a query is provided to the system, it retrieves the eight closest feature vectors in the index for each feature vector of the query. Since each feature vector points back to its source image (hence the “inverted-file” nomenclature), closeby images can be traced back and receive votes. Once all votes are attributed and counted, the images are sorted from the most to the least voted, generating the desired image rank as output. Once more, we are making this experimental setup available to the community at https://git.io/JcZGo.

More details of the methods used in the tasks above are presented in the supplementary material S1 of this manuscript. The following two subsections explain the algorithms herein proposed to perform copy-move detection and provenance analysis over scientific images, respectively. These are the tasks within SILA that received most of our original contributions.

### Copy-move detection solution

**Figure 9:**
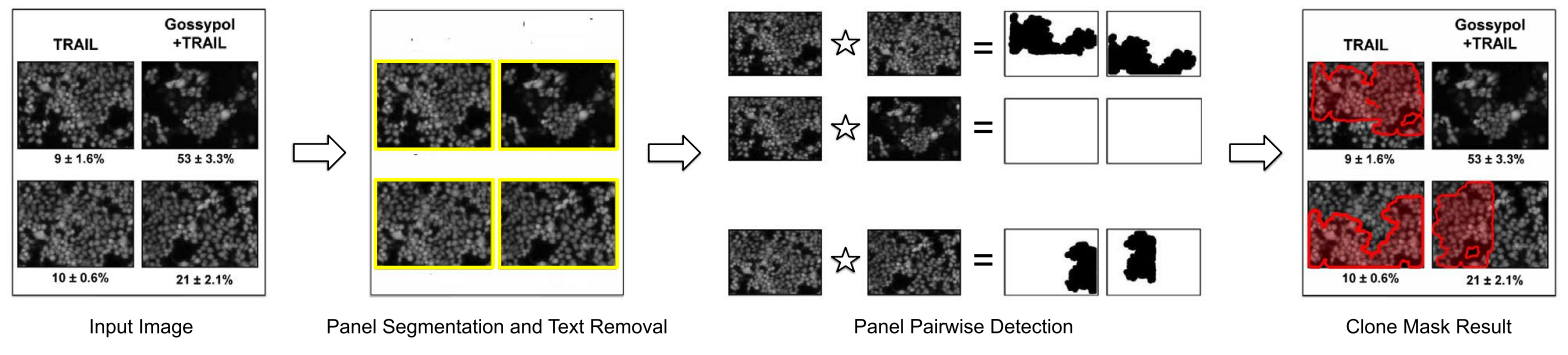
A block diagram depicting the operation of the copy-move detection solution implemented in SILA. First, a given image is segmented into sub-panels and overlaid texts are removed. The resulting sub-panels are then combined two-by-two, leading to panel-pairwise single images submitted to the copy-move detector. After all the possible sub-panel pairs are processed, the multiple resulting cloned-region masks are combined, leading to the final clone mask result. All original panels within this figure belong to a paper published as Creative Commons content, under DOI 10.1074/jbc.M110.172767.

**Figure 10:**
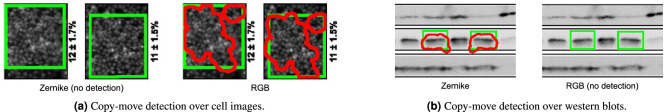
Comparison of copy-move detection results using either Zernike or RGB features. Ground-truth annotation is represented by green boxes, while the red regions correspond to the cloned content automatically detected by SILA. In (**a**), detection over cell images. In (**b**), detection over western blots. While Zernike features did not work for cell images, RGB features did not work for western blots. For this reason, we propose a fusion of such techniques within SILA. All original panels within this figure belong to papers published as Creative Commons content, under DOI (**a**) 10.1074/jbc.M110.172767 and (**b**) 10.1074/jbc.M803547200. Here, they were cropped, enlarged, and annotated over, in either green or red, to convey the findings of SILA.

Many algorithms were proposed for copy-move forgery detection in the literature. However, none of them was designed to handle the detection of cloned content within scientific images properly. As we show in Fig. 9 (Input Image, and Panel Segmentation and Text Removal), scientific images are often composed of multiple sub-components (panels) and text describing the content. To avoid false alarms due to text matching and analyze all the existing sub-panels within an image, our solution uses the panel segmentation step and includes an Optical Character Recognition (OCR) system to localize and remove text. Once panels are cleaned from text and extracted, the copy-move detector examines all the possible pairs of panels inside each figure, looking for visually similar content (see Fig. 9, Panel Pairwise Detection). Each analyzed pair thus leads to the generation of a pair of cloned-region masks. Based on the original location of the extracted panels, the multiple masks are combined at the end of the process (see Fig. 9, Clone Mask Result) to generate the final image clone mask.

Concerning the panel pairwise copy-move detection step, SILA leverages dense-field copy-move approaches^36^. Compared to other algorithms, the dense-field-based ones have the advantage of handling both additive (when entire objects are duplicated) and occlusive copy-move operations (when uniform and background small portions of pixels are duplicated to hide content). More specifically, inspired by the work of Cozzolino et al.^55^, we rely on a modified version of PatchMatch^56^, which is an efficient dense-field method for finding similar patches within an image. It thus includes a dense feature extraction step that uses Zernike moments to be robust to distortion and rotation operations, a randomized iterative algorithm for computing nearest neighbor fields, and a post-processing procedure based on dense linear fitting and morphological operations.

Nonetheless, our approach adds extra steps to adapt to the peculiar nature of the images extracted from scientific papers, which largely differ from natural scenes, especially given the presence of uniform backgrounds, lower resolution, and limited range of pixel intensity values. Figures with western blots, for example, span a small range of pixel values, whose blots are very similar in appearance, even if they are not the same. As a consequence, many false-alarm matches arise through the images, needing mitigation. To reduce these random false matches, we introduce an additional constraint to the matching procedure: matching between two regions must hold both ways. This means that a region *A* is declared forged and a copy of a region *B* only if two compatible matches are found: the match from *A* towards *B* and vice-versa.

Lastly, besides the Zernike features, we also consider the Red, Green, and Blue (RGB) values of the image pixels. By considering the three-color channels of the images, the solution turns out to be more robust even to small changes in pixel intensity values. This is shown in Fig. 10, where we notice the approach including Zernike features fails in the case of cell images (see Fig. 10a) but works in the case of western blots (see Fig. 10b), even in the presence of mirrored blots. The strategy based on RGB data, on the contrary, is successful in the case of the cell images but misses the western blots. To take the best of both worlds, SILA uses a fusion of them. Experiments comparing the results of different copy-move methods and configurations are reported on the supplementary material S1 that accompanies this manuscript. We are also making the copy-move detection experimental setup available at https://git.io/JcZGR.

**Figure 11:**
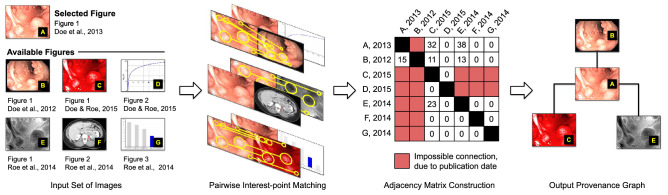
A block diagram depicting the operation of provenance analysis performed by SILA. First, a selected figure and a set of available figures extracted from different papers of interest are fed to the provenance analysis container. Next, geometrically consistent interest-point matching is performed over pairs of figures, aiming at finding similar regions between every two of them. The figure pairwise amounts of matching interest points are then used to construct an image adjacency matrix, whose connections must observe the publication dates of the papers of the figures. A figure published on a particular date cannot be connected to another one with an earlier publication date (hence the red positions within the adjacency matrix). In the end, a maximum spanning tree is computed from the adjacency matrix, leading to the output provenance graph. All original images within this figure are in the public domain^24,25^.

### Provenance analysis solution

In the particular case of figures coming from scientific publications, we developed a combination of the solutions introduced by Moreira et al.^21^ and Bharati et al.^31^ to construct provenance graphs. This approach is illustrated in Fig. 11. Given a selected suspect figure and a set of available figures of interest (which might have been previously retrieved by the task of image ranking), the system describes each one of them through 2000 RootSIFT^51^ interest points, ignoring the regions with overlaid text, to avoid further false-positive content matching (one of the novelties introduced by SILA). Similar to the case of copy-move detection, we rely on an OCR algorithm to perform text detection. Once all the interest points are extracted from each figure, we compute the geometrically consistent interest-point matches between each pair of them, following the same approach devised by Moreira et al.^21^. The number of interest-point matches established between each pair of figures is thus used to construct an adjacency matrix, whose objective is to register the similarities between the images. Here, the subjacent idea is that the more visually similar two figures are, the more interest-point matches they will share.

Additionally, inspired by the work of Bharati et al.^31^ and considering that the figures come from papers that are rich in metadata, we proposed to rely on the publication dates of the papers to constrain the adjacency matrix by avoiding connections from later to earlier published figures (based on their respective papers’ publication dates). This is somehow represented through the red positions of the adjacency matrix depicted within Fig. 11 (Adjacency Matrix Construction). As another novelty implemented by SILA, these positions cannot be used as edges of the provenance graph since they transgress the publication date order rule. Finally, Kruskal’s maximum spanning tree algorithm^57^ is used to convert the adjacency matrix to the output provenance graph (following the approach previously experimented with by Moreira et al.^21^). For the sake of reproducibility, we are making the provenance analysis experimental setup available at https://git.io/JcZGl.

## Supplementary Information

Supplementary Information 1.
