# Supplementary material for: SILA: a system for scientific image analysis
Source: Sci Rep. 2022 Oct 31;12:18306. doi: 10.1038/s41598-022-21535-3 (PMC9622827; doi:10.1038/s41598-022-21535-3)
Supplement: Supplementary file 1 — Supplementary Information 1. [file 41598_2022_21535_MOESM1_ESM.pdf]

Supplementary Material of
SILA: A System for Scientific Image Analysis

This document supplements the content of the manuscript entitled “SILA: A System for Scientific Image Analysis”, by providing more details of the proposed system architecture, as well as of each one of the five tasks that constitute the proposed system workflow.

1. SYSTEM ARCHITECTURE

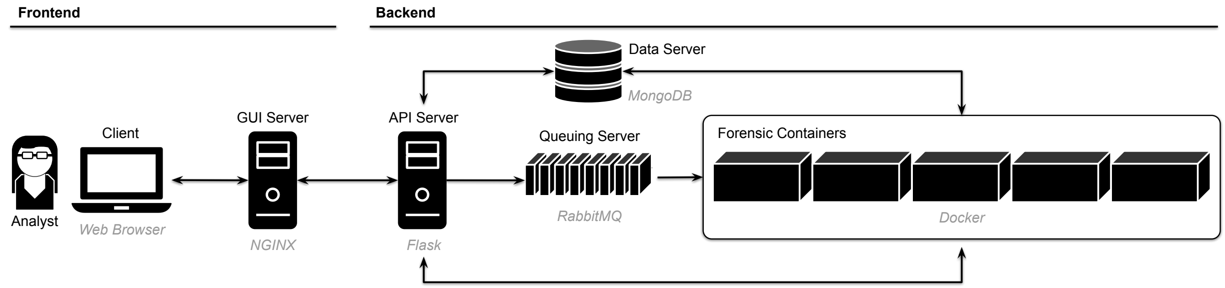

Fig. S1. Architecture of SILA. We adopted a client-server model, with frontend components focused on usability, and backend components focused on scalability and extensibility. New forensic solutions can be easily added to the system if they are made available as Forensic Containers. Labels in italic on the bottom of the components detail the technology used in SILA to implement them. Arrows express the data flow between components.

Fig. S1 depicts the architecture of SILA, properly divided into frontend and backend components. In the frontend, our main goal was usability. Within it, the central piece is the Graphical User Interface (GUI) Server, whose aim is to provide an intuitive interface that most people are familiar with. Therefore, we proposed the usage of a web-based GUI, with rich input and output graphical components (such as web-pages, click buttons, selection boxes, etc.). These components can be rendered on the client side by the currently popular web browsers, with no need for the installation of specialized software by the analysts. To use the system, all they need to do is accessing the web-address of the application.

Delving into the backend piece, our main goals were scalability and extensibility. Within it, the interaction with the frontend is centralized at the Application Programming Interface (API) Server, which is the component responsible for making calls to the forensic tools available in the system. To ensure scalability, we designed the API Server to follow the Representational State Transfer (REST) architectural style [1]. For simple workloads, such as the panel segmentation of a single selected image, the API Server may trigger the forensic tool of interest directly, by making a stateless call to it. For more complex scenarios, such as the copy-move detection of hundreds of images, we designed the use of the Queuing Server, a middleware responsible for managing the various requests from active clients and arbitrating them to the proper available Forensic Containers.

Talking about containers, they represent the tool we used to ensure extensibility. Hence, we implemented each task from the proposed workflow as an independent Forensic Container, a standalone executable piece of software that contains everything needed to run it, including libraries, configurations, and even specific operating-system environments. By using tools such as Docker [2], novel and better forensic tools can be easily added to the system, as long as they are bundled as a container.

Lastly, we added a Data Server to the application, which is responsible for persisting all the data either ingested to (such as the questioned paper PDF files), or generated by the system (such as the extracted images, captions, panels, manipulation masks, and provenance graphs). The Data Server is a resource shared among the API Server and the Forensic Containers.

2. CONTENT EXTRACTION

Here we provide more details of content extraction through its two constituent sub-tasks, namely (i) Image Extraction and (ii) Image Caption Extraction.

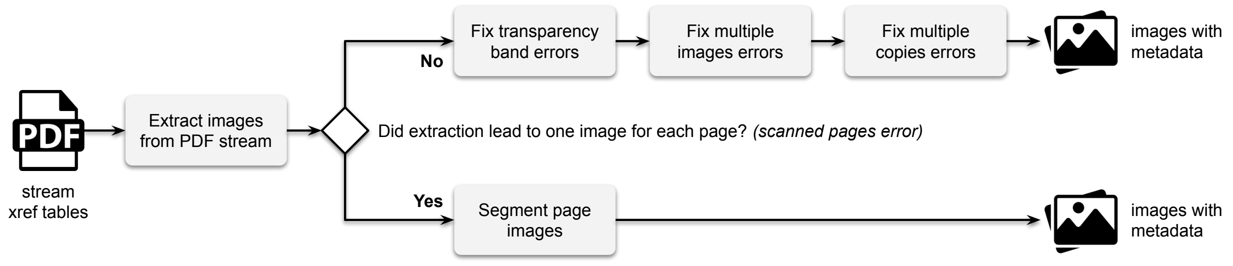

Fig. S2. Image extraction workflow. The entire process is automatic and starts from a given PDF file and ends with a set of images and their respective metadata. Possible errors due to an irregular PDF generation process are mitigated through the extraction tasks depicted as rounded rectangles.

A. Image Extraction

Metric details: To evaluate the quality of SILA's image extraction solution, we extracted images from a selection of PDF files that contained the original figures provided by the respective publishers' websites (it is common practice for scientific publishers to provide downloadable high-quality versions of figures from papers on their websites). The idea was to report how many of the original images were successfully obtained with the system. This metric is called image recall ($IR \in [0..1]$, see Eq. S1). As one might observe, it was computed based on the total numbers of available and of effectively matched original figures, and we wanted it as close to one as possible.

$$IR = \frac{\#matched\_original\_figures}{\#available\_original\_figures} \quad (S1)$$

To compute IR, we needed to compare pairs of images (with one coming from the publisher's website and the other coming from SILA's extraction module), and decide if they represented the same content (*i.e.*, they were a match). Rather than doing this manually, we adopted an automatic approach where we found, for each publisher's website image, the most similar one extracted by SILA from the same paper, based on a pairwise image similarity score S. Two images were a match if their score S was above a threshold τ_S. Considering that matching figures often present differences in resolution and translation (since the original figures are usually provided in the publisher's website at a higher resolution, with differences in the outside borders and cropping place), we selected a similarity metric that is well-known for being invariant to scaling and translation. Namely, we computed S as the Complex-Wavelet Structural Similarity (CW-SSIM) index [3]. Moreover, based on empirical analyses, we chose $S > \tau_S = 0.7$.

Operation details: Files in Portable Document Format (PDF) comprise binary streams of objects such as text, annotations, fonts, images, etc. (*i.e.*, the body of the document), and cross-reference tables (*i.e.*, Xref tables) whose purpose is to index these objects. As a consequence, PDF image streams can be found through the Xref tables, which define the position, compression type, format, and other aspects of each embedded image. In the case of SILA, we proposed to rely on the Xref tables to extract the embedded images directly from the PDF stream.

While extracting images from a series of PDF streams, we have occasionally found four types of problems that impair the quality of the images, often rendering them unsuitable for further analysis. The first one (transparency band error) is related to images embedded with an additional transparency band. In such cases, instead of obtaining a single image, two figures are wrongly extracted from the stream, both individually incomplete but with complementary appearance. We fixed this issue by automatically combining their content into a single element.

In a similar fashion, the second problem (multiple images error) occurs when single images are embedded, for whatever the reason, as multiple objects, each one containing a different portion of the original figure. We fixed this by automatically concatenating the portions into a single image.

The third problem (multiple copies error), in turn, happens due to a faulty PDF generation, when the same image is embedded many times on the same page position, with overlaps that go unnoticed in regular PDF content rendering. As a consequence, one single image leads to various copies of itself in a typical extraction process. We fixed this problem by keeping only one of them.

Lastly, the fourth problem (scanned pages error) refers to PDF documents generated from scanned documents, whose entire pages are embedded as single images. As a result, a PDF document with n pages is actually encoded as n images, one for each page. In such cases, we developed an additional routine that detects this situation and, instead of applying a typical Xref-table-based image extraction, it executes a vision-based page segmentation similar to [4].

Fig. S2 summarizes the image extraction process proposed in this work. The workflow starts with a given PDF file and ends with a set of extracted images and their respective metadata. For each obtained image, the metadata consist of the number of the PDF page containing the image, as well as the (x_0, y_0, x_1, y_1) bounding box that encloses its content.

B. Image Caption Extraction

Metric details: Two metrics were used to assess the integrity of the extracted captions, by comparing them with their respective ground-truth image-wise counterparts. On the one hand, normalized Levenshtein distance [5] ($LD \in [0..1]$) was used to measure how different ground-truth and extracted captions were at the character level. We wanted LD as close to zero as possible, since it is a distance. On the other hand, BERTScore [6] ($BS \in [0..1]$) was used to measure the semantic similarity between ground-truth and extracted captions, through the SciBert word embedding model [7] (given it was trained with scientific text examples). We wanted BS as close to one as possible.

Operation details: SILA retrieves captions from the PDF text and associates them to the images obtained during image extraction. To do so, the system extracts all paragraphs from the PDF stream with their respective metadata. Similar to the case of images, paragraph metadata comprise the text page number and (x_0, y_0, x_1, y_1) page bounding box. To select only image captions, we considered only the paragraphs that started with keywords such as “Figure”, “Fig.”, or other strings properly set in the system’s configuration. To associate images and captions, we relied on the matching of image and caption metadata. To cope with the large variety of scientific paper layouts, we adopted two simple and yet robust rules to associate images and captions. Namely, (i) an image and its caption should be on the same page (*i.e.*, they should have the same metadata page number), and (ii) they should be as close as possible in the page’s Euclidean object-placement space, with respect to their bounding boxes. A greedy algorithm was used to attribute one caption to each image, following these rules.

3. PANEL SEGMENTATION

Metric details: To measure the quality of the panel segmentation module, we used the intersection over union score ($IoU \in [0..1]$, see Eq. S2) to compare ground-truth segmentation masks (gt_mask) and the respective panels provided by the segmentation solution (sl_mask), at the pixel level. As expected, the higher the IoU value, the better the solution.

$$IoU = \frac{\#white\_pixels \in \{gt\_mask \cap sl\_mask\}}{\#white\_pixels \in \{gt\_mask \cup sl\_mask\}} \tag{S2}$$

4. IMAGE RANKING

Metric details: By comparing computed and ground-truth image ranks, we reported the performance of image ranking in terms of precision at the top-N retrieved images ($P@N$, with $N \in \{1, 5, 10\}$, see Eq. S3), averaged over a selection of images. The idea was to assess the SILA’s image ranking module as a recommender subsystem: the higher the precision values, the better the recommendations.

$$P@N = \frac{\#relevant\_images\_among\_N}{N} \tag{S3}$$

Operation details: Previous works have tried to classify images from scientific papers [8–10], but a consensus on the classes needed to organize elements (such as charts, diagrams, illustrations, etc.) has yet to emerge. This problem gets even harder when one takes into consideration the different scientific disciplines and their specific needs, ranging, for instance, from Mathematics (with need to support equations) to Medicine (with need to support specialized image acquisition like magnetic resonance imaging), and beyond to Biology (with need to support a variety of types of microscopy). Aiming to develop a more general-purpose workflow that fits different domains, we replaced the classification approach with a visual similarity one. Given an image of interest, namely the *query*, and a set of images previously extracted from a selection of PDF files, namely the *gallery*, SILA sorts the gallery elements from the most to the least similar to the query, regardless of their type, to present to the analyst.

Fig. S3 depicts an example of the ranked images ideally retrieved for a given query. To accomplish this task, we relied on techniques from the field of Content-Based Image Retrieval (CBIR). Typical CBIR solutions reduce the semantic gap between the image pixel values and the system’s purpose through a multi-level representation of the images. In the case of SILA, the purpose was to sort gallery images, preferably retrieving exact copies and near-duplicates in the first positions, followed by semantically similar images, followed by unrelated content. We wanted exact copies and near-duplicates first because they constitute key elements in the process of detecting image reuse and splicing, since they come from the same imaging pipeline of the query. Semantically similar images, on the contrary, are visually similar to the query (*e.g.*, pictures of the same type of cell), but come from different devices or capture points. Their retrieval over unrelated images is still desired, though, since they may help the analyst to spot problems such as idea plagiarism.

Given our preferences for near-duplicates over semantically similar images, we chose matching local features (*i.e.* interest points) in the lowest levels of CBIR representation. Contrary to global features such as perceptual hashing [15], which embed the entire image content as a single feature vector, local features aim at detecting and describing multiple regions of interest within the image content, which are tolerant to color and spatial transformations (*e.g.*, rotations, scaling). As a consequence, whenever the previous step of image content extraction was executed, all the resulting images were immediately described with multiple local features, which were then indexed with an inverted-file approach [16] (*i.e.*,

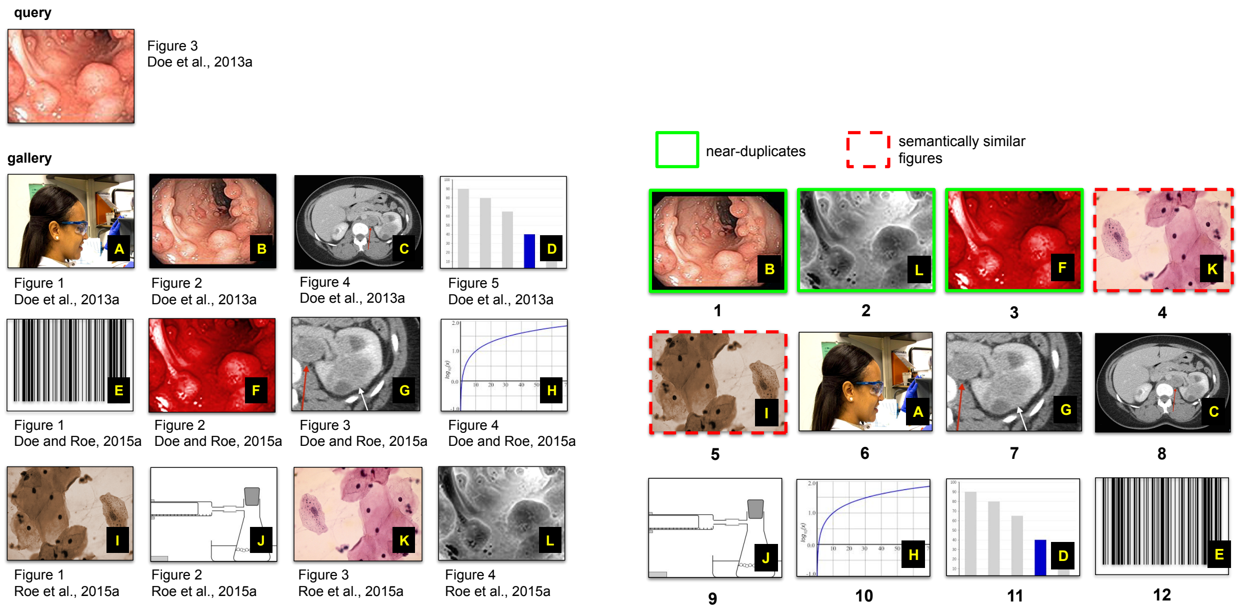

(a) Selected query and available image gallery.

(b) Desired image ranking.

Fig. S3. Designed output for image content ranking. These examples were artificially created for illustration sake. In (a), we have a suspect panel selected by the analyst to be the query ("Figure 3", obtained from Doe et al., 2013a), and the available gallery (*i.e.*, a set containing images from A to L extracted from three different papers). In (b), we have the desired ordered set of images (image ranking) related to the query, from positions 1-12. Near-duplicates, with green contiguous borders, are expected to occupy the first positions of the rank. Semantically similar images, with red dashed borders, are expected to follow near-duplicates. Unrelated content, in turn, should occupy the last positions. All original images within this figure are in the public domain [11–14].

each multi-dimensional feature vector was entirely stored with the identification number of its source image). This inverted-file index constituted the gallery information.

During content ranking, whenever a query was selected, each one of its feature vectors was used to retrieve the k-nearest gallery feature vectors, within the n-dimensional space they constituted. Since each gallery vector pointed back to its source image, close gallery images could be traced back and receive two types of votes: (i) one based on the position $p \in [1, k]$ of the gallery feature within the k retrieved features, and (ii) one based on the distance $d \geq 0$ of the gallery feature to the query feature. The position-based votes were weighted by the inverse of p (*i.e.*, the smaller the value of p, the more relevant the vote to the respective gallery image). In a similar fashion, the distance-based votes were weighted by the inverse of d. Once all the query feature vectors were processed, votes were summed and min-max normalized in a gallery-image-wise manner, for each one of the two vote types. Consequently, each gallery image received two votes, namely $V_{pos} \in [0..1]$ (related to p) and $V_{dist} \in [0..1]$ (related to d). These votes were then linearly combined into a single one, allowing for a further sorting of the gallery images from the most to the least voted. This sorted list became a query-wise image rank, which was the desired output of the image ranking task.

5. COPY-MOVE DETECTION

Metric details: To compare the ground-truth manipulation masks and copy-move detection masks computed by SILA, we followed the setup proposed in [17]. Therefore, we relied on the pixel-wise F_1-score metric, defined as:

$$F_1 = \frac{\#TP}{\#TP + 0.5 \times (\#FP + \#FN)}. \quad \text{(S4)}$$

In such case, $\#TP$ was the number of corresponding pixels over both masks that were accordingly white; $\#FP$ was the number of corresponding pixels that were black on the ground-truth mask but white on the solution-based one; and $\#FN$ was the number of corresponding pixels that were white on the ground-truth but black on the solution-based mask. By definition, we wanted $F_1 \in [0..1]$ to be as close to one as possible.

Literature comparison: In Table S1, we report the results of copy-move detection over the same scientific images obtained by different approaches from the forensic literature. Within this group, there were methods specialized

Details	F_1-score	Method
Methods based on noise analyses.	0.081	ELA [18]
	0.128	Noise-based [19]
	0.101	Splicebuster [20]
Methods based on content matching.	0.164	SIFT-based [21]
	0.202	EDF [17]
	0.149	FE-CMFD-HFPM [22]
Methods based on deep learning.	0.092	BusterNet [23]
	0.065	CMSD/STRD [24]
Proposed methods.	0.296	Basic Zernike (no panel segmentation)
	0.307	Zernike
	0.325	RGB
	**0.345**	**Zernike + RGB**

Table S1. Copy-move detection results of different forensic techniques. The herein proposed approaches obtain the best results over scientific images. SILA uses the last method within its copy-move detection module.

in highlighting noise inconsistencies [18–20], methods that tackled the problem of copy-move detection with classic content-matching approaches such as sparse or dense local descriptors [17, 21, 22], and more recent methods relying on deep-learning-based strategies [23, 24]. As one might observe, all the methods presented performance far from those belonging to our proposed solutions (represented by the last four rows of Table S1). Indeed, the less effective ones were those based on either noise residuals (which were probably not suitable for low-resolution images such as the ones published in scientific papers), or deep learning (which were trained only on natural-scene images). Better results were instead achieved by methods that used interest points and block-based local descriptors devised for visual content duplication detection. Nonetheless, even these results were below the performance of our approaches.

Ablation study: We carried out an ablation study to better understand the relevance of some steps included in our copy-move detection algorithm. First, we considered a basic version relied upon Zernike moments, which did not include the panel segmentation step and the sub-panel pairwise matching strategy (namely, Basic Zernike within Table S1). After that, we considered two modified versions, which both included the panel segmentation and sub-panel pairwise analysis steps proposed in this work. One was based on Zernike moments (namely, Zernike), and the other was based on the data from the image RGB channels (namely, RGB). Both imposed improvements over the results obtained with Basic Zernike. Moreover, while using Zernike features and RGB data provided almost comparable results individually, their fusion boosted performance (see Zernike + RGB), resulting in the best copy-move detection image masks. This is the approach currently adopted by SILA.

6. PROVENANCE ANALYSIS

Metric details: To evaluate the SILA's capabilities of building provenance graphs, we followed the experimental setup proposed in [25]. Therefore, to compare a ground-truth provenance graph $G'(V', E')$ and its corresponding graph $G(V, E)$ computed by SILA, we used the harmonic mean of precision and recall (*i.e.*, the F_1-score) respective to two key concepts, namely (i) the obtained image *vertices* V and their V' counterparts, and (ii) the established *edges* E and their E' counterparts.

The first metric, focused on comparing the vertices, is called vertex overlap ($VO \in [0..1]$):

$$VO(G', G) = \quad 2 \times \frac{|V' \cap V|}{|V'| + |V|}, \tag{S5}$$

and we wanted it as close to one as possible. The second one, focused on comparing the edges, is called edge overlap ($EO \in [0..1]$):

$$EO(G', G) = \quad 2 \times \frac{|E' \cap E|}{|E'| + |E|}, \tag{S6}$$

and we also wanted it as close to one as possible. Additionally, we also computed the $F1$-score of retrieving both vertices and edges simultaneously, dubbed vertex and edge overlap ($VEO \in [0..1]$):

$$VEO(G', G) = \quad 2 \times \frac{|V' \cap V| + |E' \cap E|}{|V'| + |V| + |E'| + |E|}. \tag{S7}$$

We computed these three metrics for each available (G', G) pair and reported their average value. In summary, these metrics assess the overlap between each G and corresponding G' provenance graph. Lastly, in the particular case of EO and VEO, we adopted the undirected-graph setup [26], where an edge within E is considered a hit whenever there is a homologous edge within E' that connects equivalent vertices, regardless of the edges' orientations.

REFERENCES

1. R. Fielding, R. Taylor, J. Erenkrantz, M. Gorlick, J. Whitehead, R. Khare, and P. Oreizy, "Reflections on the REST architectural style and "principled design of the modern web architecture" (impact paper award)," in *ACM Joint Meeting on Foundations of Software Engineering,* (2017), pp. 4–14.
2. Docker Inc., "What is a Container?" https://dockr.ly/2ZnYlty (2020). Accessed on Jun. 17, 2021.
3. Y. Gao, A. Rehman, and Z. Wang, "CW-SSIM based image classification," in *IEEE International Conference on Image Processing,* (2011), pp. 1249–1252.
4. E. Bucci, "Automatic detection of image manipulations in the biomedical literature," Nat. Cell death & disease **9**, 400 (2018).
5. V. Levenshtein, "Binary codes capable of correcting deletions, insertions, and reversals," Sov. Phys. Doklady **10**, 707–710 (1966).
6. T. Zhang, V. Kishore, F. Wu, K. Weinberger, and Y. Artzi, "BERTscore: Evaluating text generation with BERT," ArXiv e-prints, https://arxiv.org/abs/1904.09675 (2019). Accessed on Jun. 17, 2021.
7. I. Beltagy, K. Lo, and A. Cohan, "SciBERT: A pretrained language model for scientific text," ArXiv e-prints, https://arxiv.org/abs/1903.10676 (2019). Accessed on Jun. 17, 2021.
8. A. Lagopoulos, N. Kapraras, V. Amanatiadis, A. Fachantidis, and G. Tsoumakas, "Classifying biomedical figures by modality via multi-label learning," IEEE J. Biomed. Heal. Informatics **23**, 2230–2237 (2019).
9. B. Cheng, R. Stanley, S. Antani, and G. Thoma, "Graphical figure classification using data fusion for integrating text and image features," in *IEEE International Conference on Document Analysis and Recognition,* (2013), pp. 693–697.
10. R. Futrelle, M. Shao, C. Cieslik, and A. Grimes, "Extraction, layout analysis and classification of diagrams in pdf documents," in *IEEE International Conference on Document Analysis and Recognition,* (2003), pp. 1007–1013.
11. B. Shuch, "HLRCC renal tumor," https://visualsonline.cancer.gov/details.cfm?imageid=10657 (2016). Accessed on Aug. 3, 2022.
12. M. Rodriguez-Bigas, "FAP polyps - endoscopic," https://visualsonline.cancer.gov/details.cfm?imageid=10067 (2015). Accessed on Aug. 3, 2022.
13. National Cancer Institute, "Diethylstillbestrol (DES) cervix," https://visualsonline.cancer.gov/details.cfm?imageid=7630 (1983). Accessed on Aug. 3, 2022.
14. D. Sone, "DNA genotyping and sequencing," https://visualsonline.cancer.gov/details.cfm?imageid=11186 (2017). Accessed on Aug. 3, 2022.
15. C. Zauner, M. Steinebach, and E. Hermann, "Rihamark: perceptual image hash benchmarking," in *SPIE Media Watermarking, Security, and Forensics,* (2011), pp. 1–15.
16. I. Witten, A. Moffat, and T. Bell, *Managing gigabytes: compressing and indexing documents and images* (Morgan Kaufmann, San Francisco, US, 1999).
17. D. Cozzolino, G. Poggi, and L. Verdoliva, "Efficient dense-field copy-move forgery detection," IEEE Transactions on Inf. Forensics Secur. **10**, 2284–2297 (2015).
18. N. Krawetz, "A picture's worth... Digital Image Analysis and Forensics," Online document, https://www.hackerfactor.com/papers/bh-usa-07-krawetz-wp.pdf (2007). Accessed on Jun. 17, 2021.
19. S. Lyu, X. Pan, and X. Zhang, "Exposing region splicing forgeries with blind local noise estimation," Springer Int. J. Comput. Vis. **110**, 202–221 (2014).
20. D. Cozzolino, G. Poggi, and L. Verdoliva, "Splicebuster: A new blind image splicing detector," in *IEEE International Workshop on Information Forensics and Security,* (2015), pp. 1–6.
21. I. Amerini, L. Ballan, R. Caldelli, A. Del Bimbo, L. Del Tongo, and G. Serra, "Copy-move forgery detection and localization by means of robust clustering with j-linkage," Elsevier Signal Process. Image Commun. **28**, 659–669 (2013).
22. Y. Li and J. Zhou, "Fast and effective image copy-move forgery detection via hierarchical feature point matching," IEEE Transactions on Inf. Forensics Secur. **14**, 1307–1322 (2018).
23. Y. Wu, W. Abd-Almageed, and P. Natarajan, "Busternet: Detecting copy-move image forgery with source/target localization," in *Springer European Conference on Computer Vision,* (2018), pp. 168–184.

24. B. Chen, W. Tan, G. Coatrieux, Y. Zheng, and Y. Q. Shi, "A serial image copy-move forgery localization scheme with source/target distinguishment," IEEE Transactions on Multimed. pp. 1–12 (2020).
25. A. Yates, H. Guan, Y. Lee, A. Delgado, D. Zhou, T. Kheyrkhah, and J. Fiscus, "Media Forensics Challenge 2019 Evaluation Plan," https://www.nist.gov/system/files/documents/2019/03/12/mfc2019evaluationplan.pdf (2020). Accessed on Jun. 23, 2021.
26. D. Moreira, A. Bharati, J. Brogan, A. Pinto, M. Parowski, K. Bowyer, P. Flynn, A. Rocha, and W. Scheirer, "Image provenance analysis at scale," IEEE Transactions on Image Process. **27**, 6109–6123 (2018).
